# Lower Extremity Strength and the Range of Motion in Relation to Squat Depth

**DOI:** 10.1515/hukin-2015-0007

**Published:** 2015-04-07

**Authors:** Si-Hyun Kim, Oh-Yun Kwon, Kyue-Nam Park, In-Cheol Jeon, Jong-Hyuck Weon

**Affiliations:** 1 Kinetic Ergocise Based on Movement Analysis Laboratory, Yonsei University, Wonju, South Korea.; 2 Department of Physical Therapy, Kinetic Ergocise Based on Movement Analysis Laboratory, College of Health Science, Yonsei University, Wonju, South Korea.; 3 Department of Physical Therapy, College of Medical Science, Jeonju University.; 4 Department of Physical Therapy, Graduate School, Yonsei University, Wonju, South Korea.; 5 Department of Physical Therapy, College of Tourism & Health, Joongbu University, Chungnam, South Korea.

**Keywords:** dorsiflexion, hip flexion, range of motion, squat

## Abstract

The purpose of this study was to determine which variables of the range of motion (ROM) and strength of the hip, and ankle are associated with squat depth. In total, 101 healthy subjects (64 males, 37 females) participated in the study. Outcome measures consisted of the ROM of hip flexion, hip internal rotation, external rotation, ankle dorsiflexion with an extended and flexed knee joint, and strength of the hip flexor and ankle dorsiflexor. Squat depth was measured using SIMI motion analysis software. Pearson correlation was used to determine the relationship between variables and squat depth. Multiple stepwise regression analysis was performed to determine variables associated with squat depth. The multiple regression model indicated that ankle dorsiflexion with a flexed knee and the hip flexion ROM were significantly associated with squat depth in male subjects (R^2^ = 0.435) and ankle dorsiflexion with an extended knee and dorsiflexor strength were significantly associated with squat depth in female subjects (R^2^ = 0.324). Thus, exercises to increase the ROM of the ankle dorsiflexion, hip flexion, and dorsiflexor strength can be recommended to improve squat performance. Future studies should assess an increased ROM of the ankle dorsiflexion, hip flexion, or dorsiflexor strength effect on deep squat performance.

## Introduction

Squatting is a common and popular exercise among athletes and the general public (Escamilla et al., 2001; Fry et al., 2003; McCurdy et al., 2005). In particular, it has been used to increase strength of the lower extremity muscles and the correct position is taught during squatting to minimize strain on the joints and potential injury to the low back and knees (Potvin et al., 1991; Escamilla, 2001; McCurdy et al., 2005; Kritz et al., 2009). In weightlifting and power lifting, a high flexion angle of the lower limb is frequently required, and this may induce increased musculoskeletal stress or injury to the knee (Escamilla, 2001; Hemmerich et al., 2006; Sriwarno et al., 2008; Kritz et al., 2009; Kathiresan et al., 2010; Schoenfeld, 2010).

The squat is defined as a sitting posture with dorsiflexed ankles, a deeply flexed knee and hip (Kathiresan et al., 2010) and is one of the multiple joint movements performed in a closed kinetic chain (Schoenfeld, 2010). The optimal performance pattern of the squat has been described as the hips, knees, and ankles being aligned in parallel, with no mediolateral movement, while the heels remain on the ground at all times (Kritz et al., 2009). Faulty movement patterns such as mediolateral rotation of the hip, knee alignment inside or outside the hip during the movement induce increases in the compressive and shear forces at the ankle, knee, and hip joints (Powers, 2003; Chiaia et al., 2009; Kritz et al., 2009).

Previous investigators reported that decreased strength of the hip and ankle musculature reduced the ability to stabilize the lower extremity, resulting in faulty alignment of the lower extremity such as adduction and rotation of the hip and knee valgus (Schoenfeld, 2010; Nguyen et al., 2011). During downward squatting activation of the tibialis anterior muscle is needed to initiate squatting in the upright position (Robertson et al., 2008). The rectus femoris as a hip flexor as well as a stabilizer at both a hip and a knee showed increased EMG activity in the deep squat (Dionisio et al., 2008; Robertson et al., 2008). These results reported a relationship between kinematics and muscle activation of the lower extremity during squatting, however, the relationship between squat depth and muscle strength remained unclear. Therefore, it was necessary to assess which muscles contribute the most to squat performance.

Squatting performance requires strength as well as mobility of the hip, knee, and ankle, because squatting involves multiple joints (Kritz et al., 2009; Schoenfeld, 2010). Macrum et al. (2012) demonstrated that limited ankle dorsiflexion led to decreased peak knee flexion and an increased knee valgus angle during the squat and Bell et al. (2008) showed that people with medial knee displacement during a squat exhibited tight and weak ankle muscle. In addition, Chiaia et al. (2009) reported that soccer players with limited two joint hip flexors and a hip external rotation range of motion (ROM) showed deviation of the hip, knee, and foot during a full squat and step down. These studies demonstrated effects of flexibility and strength of the hip and ankle on lower extremity kinematics and muscle activities, but there are limited studies which determine factors mainly contributing to deep squat performance.

Understanding how joint mobility and strength can affect squat depth may help in evaluating lower extremity function and direct exercise strategies for individuals with limitations in squatting. Thus, the purpose of this study was to determine variables associated with deep squat depth. We hypothesized that factors such as the ROM and strength of the hip and ankle would explain deep squat depth. We anticipated that the results of this research could be used to enhance the evaluation of the squat depth performance and highly correlated factors with squatting could be targeted as part of an intervention program to improve squat performance.

## Material and Methods

### Participants

One hundred-one subjects (male=64, female=37) were recruited as a convenience sample from students enrolled at the Yonsei University. Characteristics of the 101 subjects and descriptive statistics for the ROM and strength of the hip and ankle are presented in Tables 1 and 2, respectively. Subjects were excluded if they reported having 1) neurological signs or lower extremity chronic pain or symptoms, 2) a history of lower extremity injury within the 6 months before data collection, or 3) a history of lower extremity surgery. All data were collected in a single testing session, and all procedures were conducted on the dominant leg. The dominant leg was defined as the leg used to kick a ball twice or more among three trials (Johanson et al., 2008). Each subject was assessed by the same examiner who was a physical therapist with 3 years of clinical experience. This study was approved by the Yonsei University Wonju institutional review board. All participants provided a written informed consent statement and were supplied with information about the study design prior to participation.

### Procedure

#### Range of motion measurement

A universal goniometer (UG) with a double-armed full-circle protractor was used to measure the active ROM of the hip, and ankle joint. The UG is a useful and convenient device for ROM measurement. Passive ROM movements of the hip and ankle were performed three times prior to data collection for warm-up and familiarization purposes. For ROM measurement of hip flexion, the subject laid on a table (Johanson et al., 2008; Nussbaumer et al., 2010). The axis of the UG was placed over the greater trochanter of the femur by aligning the stationary arm of the UG with the mid-axillary line and a movable arm of the UG with the lateral epicondyle of the femur. For measurement of the hip internal and external rotation ROM, the hip and knee were flexed at 90° (Nussbaumer et al., 2010). The axis of the goniometer was centered over the patellar apex by aligning the stationary arm of the UG with the anterior superior iliac spines of the pelvis and the movable arms were aligned with the tibial shaft. The ankle dorsiflexion ROM was measured in two knee positions: ankle dorsiflexion with a full extended and 90° flexed knee, while subjects were prone on an experimental table with feet hanging off the end of the table (Johanson et al., 2008). The axis of the UG was placed over the lateral aspect of the calcaneus by aligning the stationary arm of the UG with the fibular head and the moving arm of the UG with the lateral aspect of the fifth metatarsal. All tasks were performed three times by the same investigator and data were averaged across the three trials for each subject.

#### Strength measurements

All strength measurements were performed using a hand-held dynamometer (Lafayette Manual Muscle Test System, Lafayette Inc., North Lafayette, USA). The test procedure followed the isometric “make” test of Bohannon (Bohannon, 1997). Before the test, the subject practiced for 5 min to induce maximal strength against the dynamometer plate. To measure hip flexor strength, the subject was positioned in a supine position with the hip flexed to 90° and the knee relaxed. Resistance was applied with the dynamometer just proximal to the femoral condyle (Andrews et al., 1996). For testing ankle dorsiflexor strength, the hip, knee and ankle were placed to extend fully and the foot was off the experimental table (Andrews et al., 1996). The examiner applied resistance just proximal to the metatarsophalangeal joints. The strength of each muscle was measured in a gravity-neutralized position and the dynamometer was positioned perpendicularly to the tested limb segment. Subjects were instructed to build their maximal force over a 2 s period and then maintain their maximum effort for another 5 s. During trials, a second investigator helped to stabilize the proximal segment. All tasks were performed three times and data was averaged across the three trials for each subject. Intra-rater reliability for all strength measurements was excellent (hip flexor: ICC3,1 = 0.978; SEM = 0.89 kg; dorsiflexor: ICC 3,1 = 0.988; SEM = 1.38 kg).

### Squatting: photographic and data analysis using the SIMI software

The subject was required to stand with legs apart at pelvic width, with both hands clasped and held on the back of his/her head. Subjects squatted to reach the greatest depth of the squat while continuing to look straight ahead. The squat was performed as low as possible without heel-off and the maximal squatting position was maintained for 5 s. The maximum squatting position was captured using a digital camera. To collect photographs of the sagittal plane view, a digital camera (Samsung, Korea) was placed at 2.4 m from the sagittal plane of the subjects at the height of the knee and perpendicularly to the floor using a water-based level.

Collected images were imported and analyzed using the SIMI software (SIMI Motion 5.0 Reality Motion Systems, Unterschleissheim, Germany), and squat depth was measured vertically between the hip and the floor. The squat depth of each subject was normalized using their leg length and presented as % leg length. All data were collected three times, and the average of the three trials was used for further analysis.

### Data Analysis

Pearson correlation matrices were constructed to examine the relationships between the ROM and strength of the hip, ankle and squat depth in the squatting position. To investigate which lower limb variables contributed most significantly to squatting ability, multiple regression models with a stepwise selection procedure were performed for the active ROM of the hip flexion, internal and external rotation, and ankle dorsiflexion and strength of the hip flexor and ankle dorsiflexor at the dominant leg as independent variables, while squat depth (% leg length) was the dependent variable. The determination coefficient (R^2^) shows variation in the squatting ability that is explained by the regression variables. Pearson correlation and multiple regression were conducted separately for each gender. Data analysis was conducted using the SPSS software (ver. 12.0) and the significance level was set at *p* = 0.05.

## Results

### Correlation between the ROM and strength of the hip, ankle and squat depth

**Figure 1:**
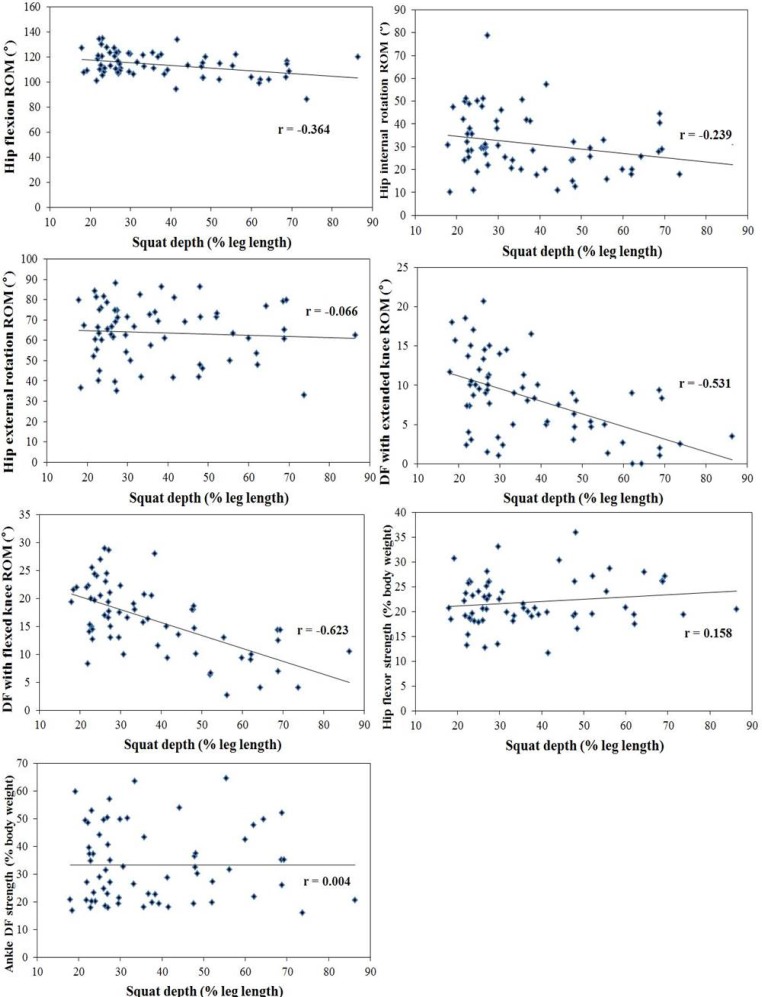
Pearson correlation coefficients between the ROM and strength and squat depth in male subjects.

**Figure 2:**
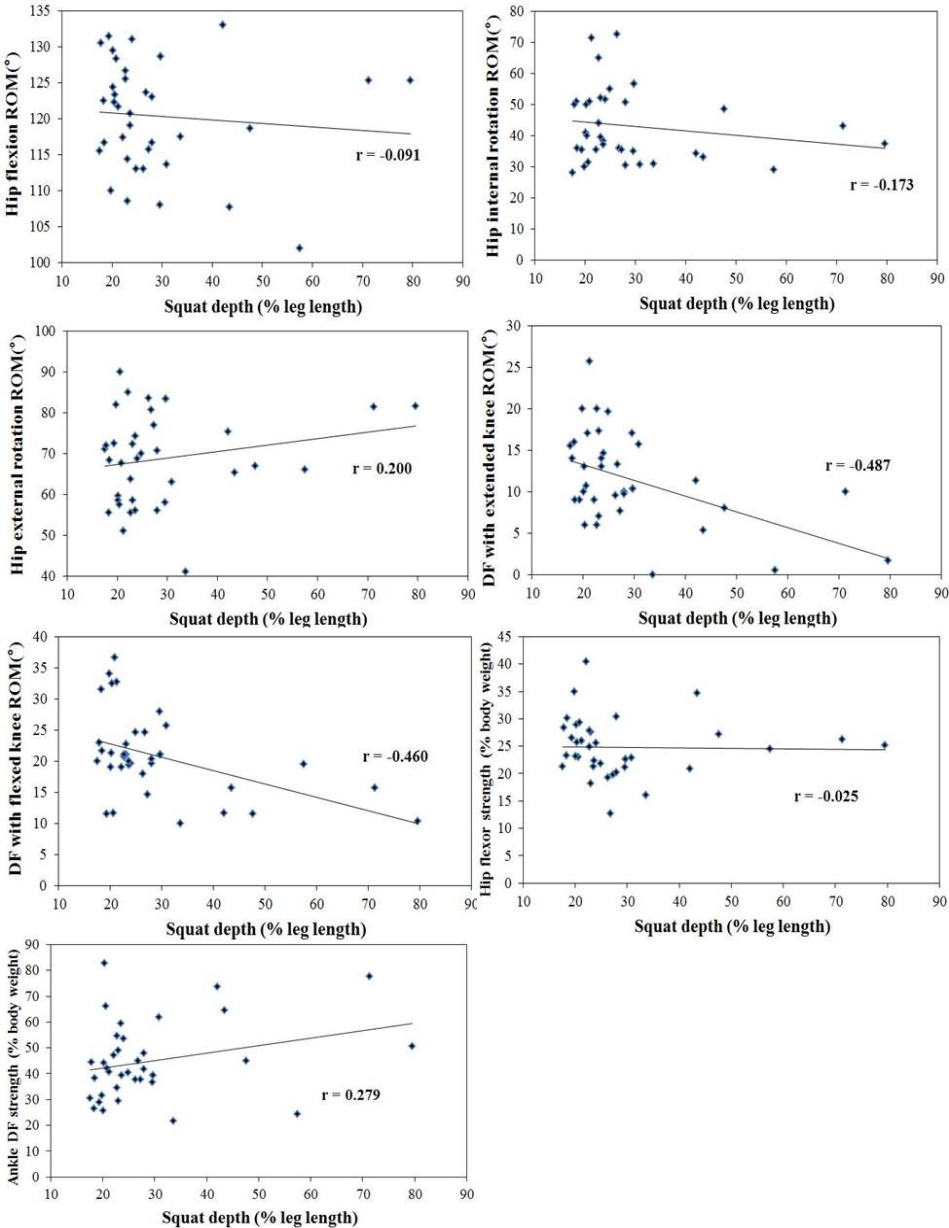
Pearson correlation coefficients between the ROM and strength and squat depth in female subjects.

Figures 1 and 2 show the correlation coefficient between the ROM and strength of the hip, ankle and squat depth in male and female subjects, ranging from fair to good. In males, there was a significantly negative correlation with the ROM of the hip flexion, internal rotation, and ankle dorsiflexion with an extended and flexed knee and squat depth (r = −0.623 to −0.239; *p*<0.05). In females, the ROM of the dorsiflexion with an extended and flexed knee was significantly negatively correlated with squat depth (r = −0.487 to −0.460; *p*<0.05), while there was a positive correlation between strength of the dorsiflexor and squat depth (r = 0.279; *p*<0.05). No significant correlation between the ROM of the hip external rotation or strength of the hip flexor and squat depth was found in the squatting position (*p*>0.05).

### Multiple regression analyses

Stepwise multiple-regression analyses were performed to identify variables that contributed significantly to deep squat depth in healthy subjects. In the results of the regression analyses for males, ankle dorsiflexion with a flexed knee and the hip flexion ROM were included as predictor variables, accounting for 43.5% of the variance in squat depth (Table 3; *p*<0.001). For females, the ankle dorsiflexion ROM with an extended knee and strength of the dorsiflexor were included as predictor variables, accounting for 32.4 % (Table 4; *p*<0.001).

## Discussion

We investigated factors associated with squat depth in healthy subjects. Deep squatting ability is important for activities on the ground and efficient exercise to increase muscle strength of the lower extremities. However, to our knowledge, there has been no previous report as to which factors are associated with squat depth. In this study, we demonstrated that the ROM of the ankle dorsiflexion with a flexed knee and hip flexion were important factors for deep squatting in males, and dorsiflexion with an extended knee and dorsiflexor strength were important factors in females. Thus, these results may help in designing exercise programs to improve deep squatting depth in subjects with poor performance.

Cook et al. (2010) reported that the deep squat test is one of the screening tools to assess bilateral symmetrical mobility of the hip, knee, and ankles. Lower extremity mobility, stability, postural control, and pelvic and core stability are necessary for safe deep squatting performance (Bell et al., 2008; Kritz et al., 2009; Schoenfeld, 2010). Previous studies have suggested that weakness and/or limited mobility in lower extremity musculature are limiting factors in performing successful deep squats (Escamilla et al., 2001; Fry et al., 2003). Thus, when an individual with decreased strength and/or limited mobility of the lower extremity performs a deep squat, faulty movement patterns and compensations are used to complete the movement pattern.

In the resulting model, the ankle dorsiflexion ROM with flexed and extended knee correlated significantly with squat depth, accounting for 38.8 and 23.7% of the variance in male and female subjects, respectively (p<0.05). Hemmerich et al. (2006) reported that the average ankle dorsiflexion angle required was 38.5 ±5.9° during a squat. Because the ankle is an important part of the closed-chain movement during deep squatting activities, limited mobility and stability of the ankle joint could inhibit performance of the proximal joints (Butler et al., 2010). Limited ankle dorsiflexion because of a tight soleus, gastrocnemius, capsular tissue, or abnormal osseous formation of the ankle can cause limited squatting and lunging movement with knee flexion or extension (Smith and Reischl, 1988; Bennell et al., 1998; Piva et al., 2005; Mauntel et al., 2013). Mauntel et al. (2013) reported that people with restricted ankle dorsiflexion showed increased medial knee displacement when compared to the subjects without restricted ankle dorsiflexion during single leg squatting. In addition, medial knee displacement (valgus) during the squatting task in people with a less ankle dorsiflexion ROM was diminished when the dorsiflexion ROM was increased by placing a wedge under the calcaneus (Bell et al., 2008). In our study, we demonstrated that the ankle dorsiflexion ROM was a major factor in deep squatting ability that affected squat depth. This suggests that squat depth can vary with ankle dorsiflexion mobility, which may be useful information when developing a treatment plan and preventing faulty movement patterns occurring due to restricted ankle joint mobility during squatting.

One of the primary roles of the hip joint is to provide a pathway for the transmission of forces between the lower extremities and pelvis during activities such as squats. During a squat, the mean hip ROM has been reported to be 95.4 ±26.6° of flexion to reach a maximal squat (Hemmerich et al., 2006). If hip flexion mobility is decreased, people may use a trunk flexion strategy to achieve desired squat depth, compensating for the decreased hip mobility. This strategy is not recommended because of the increased stress placed on the lumbar spine (Kritz et al., 2009; Schoenfeld, 2010). The results of the present study demonstrated that hip mobility was negatively correlated with squat depth in males, and decreased hip flexion mobility can be an important secondary factor to expect when the distance from the hip to floor is large. Thus, subjects who have a high squat depth need to improve hip flexion mobility for safe squatting (Woerman, 1989; Sahrmann, 2002). During hip flexion, if hip posterior structures, such as the posterior capsule and lateral rotator are stiff and/or short, the femoral head does not glide posteriorly, resulting in limited hip flexion (Sahrmann, 2002). In our study, we observed that squat depth was significantly and negatively correlated with the passive ROM of the hip internal rotation (r = −0.239; p<0.001) in males. Subjects were required to squat without motion of the hip internal or external rotation, while keeping the hip, knee, and ankle parallel during squatting. It is possible that subjects with limited hip internal rotation have difficulty in squatting. Thus, this result suggests that increasing the ROM of the hip internal rotation could help to increase squat depth.

Ankle dorsiflexor muscle strength explained an additional 8.7% of the variance in squat depth. The ankle plays an important role in the closed kinematic chain during the deep squat. Schoenfeld (2010) reported that decreased strength of the ankle musculature may induce excessive medical knee displacement and dynamic valgus. In our study, during the down squat, both hips and feet were required to align without excessive movement inside or out. Although we examined only association between ankle dorsiflexor and squat depth, it is possible that this strength deficit resulted in limitation of the deep squat to avoid mediolateral movement of the lower extremity.

Our study has several limitations. The first limitation is the lack of generalizability to other age groups; all the subjects participating in this study were in their twenties. Second, we only focused on the ROM and muscle strength in the lower extremity. Lumbar stability and thoracic mobility are also important factors to decrease compressive and shearing force of the lumbar region during deep squatting (Schoenfeld, 2010). Third, this study was a cross-sectional study and merely suggests associations between squat depth and the ROM of the hip and ankle.

## Conclusions

Squatting has been regarded as an exercise to increase the strength of the lower extremity musculatures. Although squatting performance is commonly used in training or clinical settings, there may still be unknown factors that determine squat depth. This is the first reported study to investigate factors associated with squat depth. The results of this study show that squat depth is negatively related to the ROM of the hip flexion, internal rotation, and ankle dorsiflexion with an extended and flexed knee in males and dorsiflexion with an extended and flexed knee in females. Additionally, strength of the ankle dorsiflexor is positively related to squat depth in females. The ROM of the ankle dorsiflexion is a major factor affecting squat depth in both genders, followed by the hip flexion ROM in male and ankle dorsiflexor strength in female subjects. According to sex, different factors influenced squat ability, what suggests that clinicians and athletes should approach the improvement of squat depth depending on gender. Based on our results, we recommend that in training for the deep squat, it is important to increase the ankle dorsiflexion and hip flexion ROM in male, and the ankle dorsiflexion ROM and strength in female subjects if they are limited. Improved mobility and strength of the ankle and hip joint can help in preventing injuries related to squatting and help to ensure its safe performance.

## Figures and Tables

**Table 1:** Subject characteristics (N =101)

Characteristic	Males (N = 64)	Females (N = 37)
Age; years (mean ± SD)	25.69 ± 5.93	21.95 ± 2.17
Body height; cm (mean ± SD)	174.25 ± 5.86	159.44 ± 24.50
Body mass; kg (mean ± SD)	67.61 ± 7.65	54.00 ± 6.30
Leg length; cm (mean ± SD)	88.46 ± 4.22	83.24 ± 3.61

**Table 2:** Range of motion (°) and strength (% body weight) of the hip and ankle

	Males (N = 64)	Females (N = 37)
Lower limb ROM		
Hip flexion	113.87 ± 9.76	120.34 ± 7.70
Hip internal rotation	31.32 ± 13.02	43.05 ± 11.73
Hip external rotation	63.80 ± 14.21	68.72 ± 11.27
DF with extended knee	8.23 ± 5.29	11.54 ± 5.58
DF with flexed knee	16.29 ± 6.23	20.83 ± 6.67
Lower limb muscle strength		
Hip flexor	21.99 ± 4.79	24.76 ± 5.32
Ankle dorsiflexor	33.39 ± 13.89	44.70 ± 14.85

DF: dorsiflexion, ROM: range of motion.

**Table 3:** Results of the stepwise multiple regression analysis for male subjects

Dependent variable	Model	Independent variable	*R^2^*	Adjusted R^2^	*B*	T
Squat depth (% leg length)	Model 1	DF ROM with a flexed knee	0.388	.387	−1.669	−6.265^*^
	Model 2	DF ROM with a flexed knee	0.435	.416	−1.520	−5.706^*^
		Hip flexion ROM			−.383	−2.249^*^

DF: dorsiflexion, HF: hip flexion, ROM: range of motion.

* p<0.05.

**Table 4:** Results of the stepwise multiple regression analysis for female subjects

Dependent variable	Model	Independent variable	*R*^2^	Adjusted R^2^	*B*	T
Squat depth (% leg length)	Model 1	DF ROM with an extended knee	0.237	.215	−1.242	−3.296^*^
	Model 2	DF ROM with an extended knee	0.324	.284	−1.266	−3.517^*^
		Ankle dorsiflexor strength			0.283	2.093^*^

DF: dorsiflexion, ROM: range of motion.

* p<0.05.
